# Beyond beauty: Does visual art facilitate social cognitive skills?

**DOI:** 10.1371/journal.pone.0308392

**Published:** 2024-10-04

**Authors:** Yagmur Ozbay, Suzanne Oosterwijk, Eftychia Stamkou

**Affiliations:** Department of Psychology, University of Amsterdam, Amsterdam, Netherlands; Novi Sad School of Business, SERBIA

## Abstract

Engaging with art can move individuals through a myriad of emotions, provoke reflective thoughts, and lead to new ideas. Could art also influence interpersonal outcomes pertaining to the ways we interact with others and navigate the social world, that is, our suite of social cognitive skills? Here, we focus on visual art to explore the effect of art engagement on personal aesthetic experience and social cognitive skills. Across two studies, using veridical paintings and matched non-art photos, we examined the effect of art engagement on emotional (e.g., awe, being moved) and eudaimonic experiences (e.g., reflective thoughts), as well as social cognitive skills pertaining to Theory of Mind (ToM) and recognition of other’s emotions. Further, we varied the depth with which participants engaged with the experiences of the characters in the artworks, to assess whether deep social information processing could boost the effect of art engagement on social cognitive skills. Our findings showed that art engagement altered personal aesthetic experience through changes in emotional and eudaimonic outcomes. However, we did not find any support for the effect of art engagement on social cognitive skills: Neither engaging with art, nor art in combination with deep social information processing, influenced performance on social cognitive skills of ToM and emotion recognition. The effect of art engagement on personal aesthetic experience and the absence of effect on social cognitive skills highlight the nuanced nature of individuals’ interactions with art. We discuss these results considering the varied ways of engagement with different artforms and in relation to different operationalizations of social cognitive skills.

## Introduction

“Any form of art is a form of power; it has impact, it can affect change–it can not only move us, it makes us move.” [1]

Many, like the actor and activist Ossie Davis, believed that art cannot only move individuals through unique emotional experiences, but can also change the way we see the world and others. While empirical evidence suggested that art holds the emotional end of this bargain, evoking a myriad of strong emotions and thoughts in the viewer [2–6], less attention was given to its potential impact beyond such personal experiences. Could art also influence interpersonal outcomes pertaining to the ways we interact with other people and navigate the social world?

Previous research suggests that reading literary fiction boosts social cognitive skills by triggering social information processing mechanisms, such as simulation of characters’ experiences and perspective taking [7, but see 8, 9]. One-time, brief sessions of reading have been shown to enhance Theory of Mind (ToM), understanding of others’ mental states, in adults, suggesting “ToM may be influenced by engagement with works of art” [10, p. 377]. While there is empirical support for this effect for reading literary fiction and attending theatre [11], other artforms, such as visual art, remain untested. Although shared elements and modes of engagement across the arts suggest the possibility of similar outcomes for various artforms, distinctive properties, such as the brevity of engagement that characterizes visual art experiences, or the ambiguous nature of visual narratives, may require specific consideration.

In the present studies, we focus on visual art, specifically paintings, to explore whether art engagement affects not only our personal aesthetic experience but also our social cognitive skills. We examined the effect of visual art engagement on emotional and eudaimonic experiences, and tested whether art engagement facilitated Theory of Mind (ToM) and the recognition of other people’s emotions. Moreover, we varied the depth in which participants engaged with the characters’ experiences in the artworks, to test whether the depth of social information processing boosts the effect of art engagement on social cognitive skills and influences personal aesthetic experience.

In what follows, we review how visual art engagement influences personal aesthetic experience. Then, we discuss research on the relationship between art engagement and social cognitive skills. In light of the literature, we discuss the potential for visual art, specifically engagement with paintings, to improve social cognitive skills. Finally, we present an overview of the current studies.

### Personal aesthetic experiences in response to visual art

Art triggers personal aesthetic experiences through emotional and cognitive processes. First, art elicits emotions in people. Encounters with visual art, including painting, photography and film, are shown to evoke a wide range of emotions [e.g., 4] and bodily reactions, varying from chills [12, 13] to tears [14]. A set of discrete emotions, including awe, fascination, beauty, wonder and being moved, are considered to capture the core emotional responses to great works of art [15–18], and thus labelled prototypical aesthetic emotions [4].

Second, art makes people think. Encounters with visual art can give rise to distinctive patterns of cognitive responses, associated with eudaimonic experiences. This cognitive aspect of eudaimonia is characterized by meaning making, reflection and insight [19, 20]. Meaning making and reflective thoughts in response to art can ignite a deeper understanding of life and the human condition, bringing a perspective on the psychology and social reality of others [21, 22].

Accordingly, in the current study, we examined the affective (i.e., feeling) and cognitive (i.e., thinking) components of personal aesthetic experience, which correspond to the intrapersonal effects of images on the viewer. The affective component represents the emotional experience in response to art, operationalized here by subjective reports of prototypical aesthetic emotions [i.e., beauty, awe, being moved; see 4]. The cognitive component corresponds to eudaimonic experience in response to art, represented by reflective thoughts [21]. In the next section, we shift our attention from the personal aesthetic experience to interpersonal outcomes, specifically social cognitive skills.

### Can art boost social cognitive skills?

Social cognition is the suite of abilities that concern the processing of and responding to social information. It is everything that helps us to navigate the social world, and to understand and connect with others [23]. Some scholars suggest that engagement with art, through unique components of art experiences, can enhance social cognitive skills [24, 25]. These components relate to the *content* of the artworks and the *processes* that characterize art engagement. Accordingly, potential benefits on social cognitive skills are explained in terms of these two routes, as well as their interplay during art engagement [25].

The *content* route pertains to how artworks, through fictitious worlds and character portrayals, provide concrete knowledge with regard to human psychology and social interaction. Artworks that depict social scenes are packed with social information. For instance, when viewers look at a painting they can ‘read’ the emotions of the characters through their expressions, posture, and position within the scene. Viewers can also derive information from the setting, and the way the portrayed characters interact with each other within it. Thus, art with social content presents the viewers with information about different characters and life circumstances, allowing encounters with the experiences of others [24].

The *process* route suggests that art engagement offers a kind of exercise that strengthens our social cognitive skills through the repeated engagement of social cognitive processes [24–26]. Specifically, encounters with characters through art prompts social information processing, as viewers engage in perspective taking and simulate others’ experiences and emotions [24, 26]. According to theories of embodied cognition, people represent the experiences of others by engaging the same neural structures involved in acting, feeling, and sensing in the self [27, 28]. Applying this idea to visual art, when we observe a painting, we embody the (implied) actions through activation in our own sensory-motor system. Such “vicarious experiences” allow access to the meaning of others’ behavior, and aid understanding of others’ emotions and sensations [29–34]. This way, art permits exploring and experimenting with a wide range of intentions, emotions, and emotion-evoking situations [25, 35]. As such, art engagement can bring an enhanced understanding of emotions, both our own and others’, making us better at picking up emotional cues implicitly communicated [24, 36].

A key aspect of artistic depictions is to involve an acceptable amount of ambiguity [37], that calls the viewer to engage in social information processing (i.e., perspective taking, inference making) to complete character stories and to make sense of the depiction. During this process, viewers practice social cognition through art engagement. In all, the content of art and the processes that characterize art engagement provide both the knowledge and the practice that could shift social cognitive skills [24, 36].

### The case for visual art

The idea that art engagement boosts social cognitive skills has been previously tested and supported in two domains of art, literary fiction and theatre. Attending theatre, be it “Hamlet” or “A Christmas Carol”, improved adolescents’ ability to understand others’ mental states (i.e., ToM) [11]. Similarly, a series of studies on reading literary fiction showed performance gains in ToM ability [10, 38, 39]. A meta-analysis compiling findings from studies that examined the effect of reading literary fiction found a positive, small, causal effect on social cognitive skills [7]. Nevertheless, it is unclear if the effect is limited to literature and theatre, as empirical evidence to generalize the effect to other artforms, such as visual art, is lacking.

Here, we explore the potential for engagement with visual art, specifically paintings, to enhance social cognitive skills motivated by comparisons with the tested artforms. While paintings share similarities with literature and theatre in terms of the content and process routes, differences inherent in the medium and ways of engagement may ultimately determine the effectiveness of this artform in enhancing social cognitive skills. For example, while ambiguity inherent in artworks [37] prompts social information processing as the viewer tries to make sense of the depiction, it could be argued that paintings pose greater ambiguity than literary fiction or theatre. The stories in novels or plays are more explicit and told by a narrator, whereas the stories in paintings could be made up by the viewer. For instance, Kotovych and colleagues [40] showed that increasing the number of ambiguities in a text can enhance readers’ identification with a character, resulting in a deeper connection and understanding of them. This is because ambiguity allows readers to draw more from their own experiences, bringing more of themselves in the narrative through inference-making. Thus, ambiguity may work in favor of visual arts to evoke social benefits, since the viewers have more space to bring personal experiences into the story.

Yet, there are some differences that may work against visual art in prompting the same effect as literary fiction and theatre do, namely, the brevity of visual art experiences. Smith and Smith [41] found that the viewing times for a single artwork during a visit to the museum does not exceed 30-s. This may suggest that the culturally prevalent ways of engaging with visual art are more focused on the formal elements (e.g., style, form, etc.) as compared to engaging with artwork characters and their experiences, given that the latter takes more time. If social information processing (i.e., process route) does not hold as much room in experiences with paintings, visual art engagement may not enhance social cognitive skills to an extent comparable to literary fiction and theatre, or even not at all.

### Overview of studies

We conducted two studies to examine the effect of art (versus non-art) engagement and social information processing on 1) personal aesthetic experience and 2) social cognitive skills. First, we examined how art engagement affected emotional and eudaimonic experiences. Second, we examined whether art engagement could facilitate ToM and emotion recognition skills in comparison to non-art engagement. Further, we explored if deep social information processing would boost the effect of art engagement. Here, social information processing was implemented as an additional factor targeting the process route, as this was one of the proposed mechanisms behind the effect of art engagement on social cognitive skills [25]. More specifically, our social information processing manipulation varied the extent to which participants were invited to take the perspective of the depicted characters and simulate their emotional experiences while engaging with art and non-art stimuli. The second proposed mechanism, content, was integrated in our design as a stable factor with all stimuli depicting social content (i.e., depictions of people, either solo or in interaction with others).

**Fig 1:**
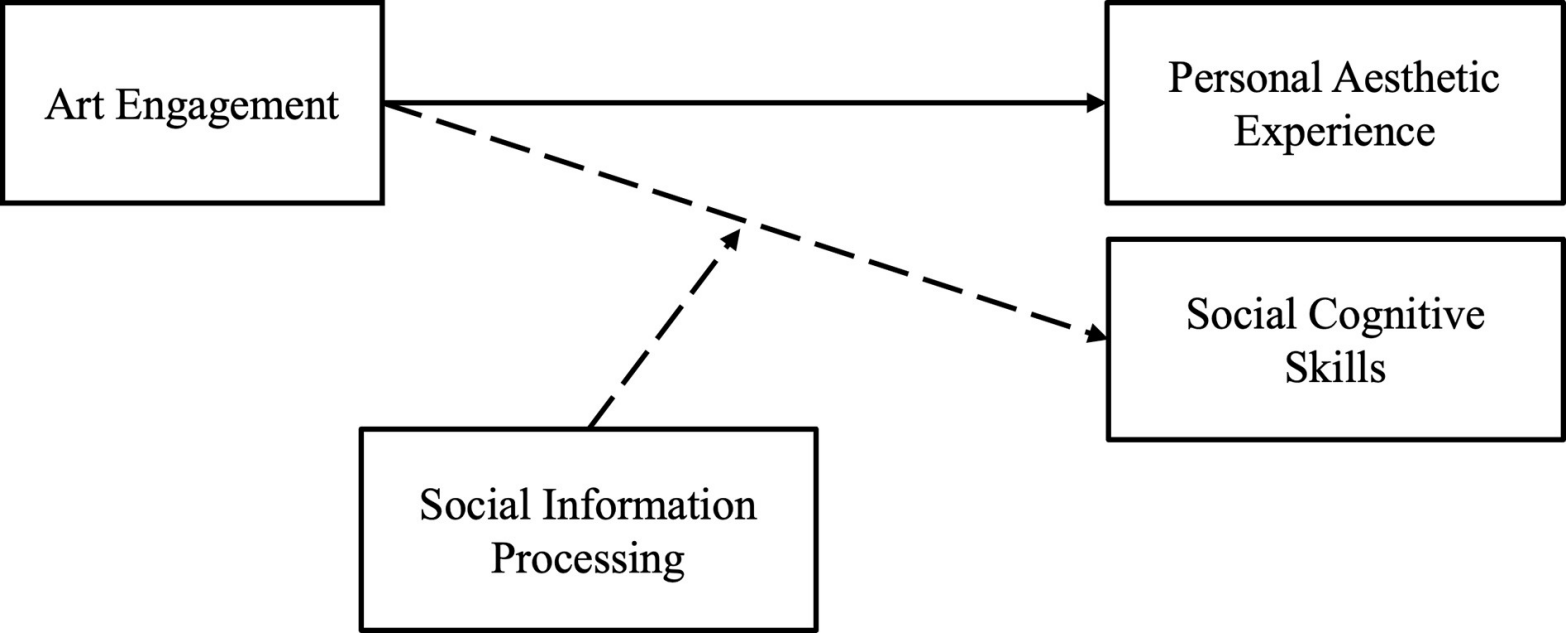
Theoretical model. The solid arrow indicates hypothesized relations and dashed arrows non-hypothesized relations.

Following the literature, we hypothesized that art engagement will evoke stronger personal aesthetic experience (emotional and eudaimonic). We did not formulate directional hypotheses on the effect of art engagement on social cognitive skills (ToM and emotion recognition). Instead, we explored the main effect of art as compared to non-art engagement on social cognitive skills, as well as the interaction effect of art engagement and social information processing. Our theoretical model is visualized in Fig 1.

We first conducted a pilot study to select and match the art and non-art stimuli (see S1 File). This formed the basis of the art engagement operationalization in the main studies. Crucially, this process was informed by previous work that examined art-elicited aesthetic experiences, and aimed to address certain methodological limitations. Namely, the use of framing manipulations as the sole distinction between art and non-art, and the lack of comparison conditions pertaining to non-art objects. Some studies that examined aesthetic responses to art prioritized experimental control by assessing ratings of the same object framed either as art or non-art. By using controlled experimental stimuli to match both categories of framing, such as IAPS pictures [IAPS; 42–45], this line of research essentially tested the “art schema effect” [46, p. 120] rather than the effect of art engagement on aesthetic experiences. Other studies that assessed emotional responses to art sought ecologically valid assessments by using artworks created by artists [47–50]. Although they expanded our knowledge about art experiences as they occur in naturalistic settings, they lacked experimental controls to show how art experiences differ from non-art experiences. In the current study, we aimed to develop a paradigm that ensured both an ecologically valid conceptualization of art experience and experimental control between respective conditions. To this end, we employed famous artworks and their visually matched photo counterparts. In addition to the surface-level visual similarity, we statistically matched art and non-art image pairs on emotional valence and social identification, and only included pairs in which the painting was rated to be significantly more artistic than the photo match. Consequently, in Studies 1 and 2, we manipulated art engagement by presenting participants with paintings framed as a collection of famous artworks or non-art photos that were visually matched to the paintings and presented as a set of pictures (see S1 File for a detailed description of the Pilot Study and the stimuli).

Across the two main studies, we manipulated social information processing by instructing participants to focus on the experiences of the characters depicted in the images. In Study 1, we operationalized social information processing by instructing participants to explicitly consider and describe the focal character’s experiences in writing versus the colors and objects present in the image. In Study 2, we standardized social information processing by presenting the images with narrative texts that provided information about the characters’ lives and inner worlds versus presenting the images without narratives.

**Fig 2:**
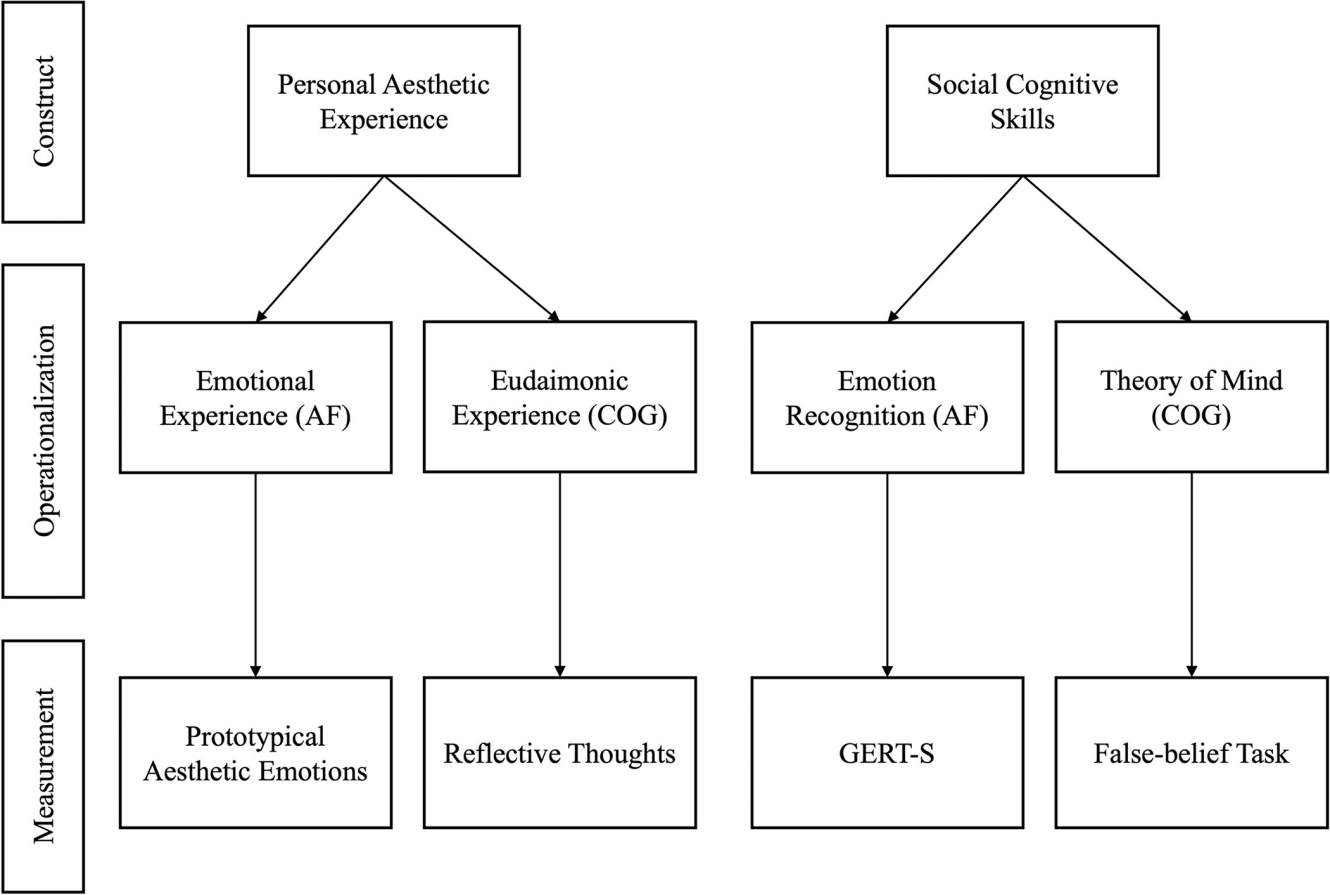
Tree-map of dependent variables and conceptualization. AF = Affective component, COG = Cognitive component.

In both studies, we measured the affective and cognitive components of personal aesthetic experience and social cognitive skills. Personal aesthetic experience was separated into emotional and eudaimonic experience, and social cognitive skills into emotion recognition and ToM. Specifically, while ToM is about mental state attributions focused on judgements about others’ intentions and beliefs [51, 52], emotion recognition is about forming accurate judgements of others’ discrete emotions [53]. We assessed both social cognitive skills through tasks that targeted *objective performance*. This is an important distinction overlooked in the literature [e.g., 7], because self-report and performance-based measures of a construct may evaluate different aspects, such as the individual’s own perception about themselves versus an objective measure of the underlying ability [54]. Here, we operationalized social cognitive skills using performance-based tasks that yield standardized scores, enabling objective comparisons across individuals. An overview of the constructs, operationalization, and measurement are displayed in Fig 2.

## Study 1

In Study 1, we tested the effect of art (vs. non-art) engagement and social information processing on personal aesthetic experience (emotional and eudaimonic) and social cognitive skills (ToM and emotion recognition).

### Method

#### Participants

A priori power analysis demonstrated that 382 participants were needed to detect a small to medium effect size (*f* = 0.17) in a 2-way ANOVA with α = .05 and power of .80. Our sample consisted of 404 participants, who were recruited through Prolific and participated online via Qualtrics. Three participants were removed from the analyses due to failing attention checks leaving a final sample of 401 (50% female, *M*_age_ = 42.8, *SD*_age_ = 13.6). Participants were recruited in April 2022, and gave written informed consent prior to participation. Data collection was anonymized, and the authors did not have access to any identifying information after the data collection. The experiment was approved by the University of Amsterdam’s Ethics Review Board (2022-SP-14826).

#### Design and procedure

Participants were randomly assigned to a 2 (art engagement: yes vs. no) x 2 (social information processing: deep vs. control) between-subjects experimental design. They were presented with 9 images, one at a time, which were either paintings or photos. This experience was framed as viewing “a collection of paintings” for the art condition or “a set of pictures” for the non-art condition. In the beginning of the art condition, participants were shown two images of an exhibition room with the upcoming artworks displayed on the walls and were told to imagine they were walking around in this exhibition.

For the manipulation of social information processing, the instructions were as follows: “All of the paintings [pictures] depict humans. For each painting [picture], try to take the perspective of the person depicted. Try to acknowledge their thoughts and feelings.” In the control condition, we asked participants to pay attention to the objects and colors present in the images. Three out of nine images were focal, where participants were instructed to either write a short paragraph about the experience of the person depicted or list the colors and objects present in the image. Viewing of the non-focal images was self-paced. For the focal images, however, participants were instructed to work on their answer for at least 40 seconds, and could not proceed to the next page sooner.

After participants viewed the whole set of images, they reported on their emotional and eudaimonic experiences. They then completed tasks assessing ToM and emotion recognition skills. Finally, they answered a question that served as manipulation check for social information processing.

*Emotional experience*. Participants rated their viewing experience on four prototypical aesthetic emotions: beauty, fascination, awe, and being deeply moved (α = .91). All items were answered on 7-point Likert scales ranging from 1 = *not at all* to 7 = *very much*.

*Eudaimonic experience*. Participants completed the Reflective Thoughts scale [21] (α = .88) containing the following four items: “The images were… thought-provoking”, “… made me think about myself”, “… inspired me to think about important issues” and “… helped me to better understand other people”. All items were answered on 7-point Likert scales ranging from 1 = *not at all* to 7 = *very much*.

*Theory of Mind (ToM)*. Following previous studies in the literature, we employed a false-belief task to measure ToM ability [see 7]. The task involved a false-belief story and seven true/false statements about beliefs and intentions of the story characters. The story was adapted from Rowe and associates [55]. One of the statements assessed participants’ memory about the story and was used for control.

*Emotion recognition*. To measure emotion recognition ability, we used the short version of the Geneva Emotion Recognition Test [GERT-S, α = .80; 56]. GERT-S consists of 42 short video clips (approx. 2-s each) in which actors express 14 different emotions through verbalizing pseudolinguistic sounds. These emotions are anger, pride, joy, amusement, pleasure, relief, interest, surprise, anxiety, fear, despair, sadness, disgust, and irritation. After seeing each clip, participants are asked to choose which emotion was expressed by the actor.

*Social information processing (SIP) manipulation check*. To assess whether the social information processing manipulation was successful, we asked participants to rate the statement: “I tried to imagine the feelings, thoughts, and reactions of the characters depicted in the images” on a 7-point Likert scale ranging from 1 = *not at all* to 7 = *very much*.

A full list of scales used in our studies is available in the S1 File. Measures that were employed to assess variables outside the focal questions of Study 1 and Study 2 are not listed here, however, they can be found in the S1 File with additional analyses.

### Results

Descriptive statistics for the manipulation check and main dependent variables are presented per condition in Table 1.

**Table 1:** Descriptive statistics of outcome variables across conditions of art engagement and social information processing in Studies 1 and 2.

	Art Engagement				Non-Art Engagement				Total^a^	
	Deep Social Information Processing		Control		Deep Social Information Processing		Control			
	*M (SD)*	95% CI	*M (SD)*	95% CI	*M (SD)*	95% CI	*M (SD)*	95% CI	*M (SD)*	95% CI
Study 1										
SIP Manipulation Check	6.11 (1.13)	[5.89, 6.33]	4.34 (1.64)	[4.01, 4.67]	5.77 (1.22)	[5.52, 6.01]	3.51 (1.69)	[3.18, 3.83]	4.32 (1.59)	[4.16, 4.47]
Emotional Experiences	4.46 (1.51)	[4.17, 4.75]	4.34 (1.47)	[4.05, 4.63]	2.96 (1.24)	[2.71, 3.21]	2.94 (1.33)	[2.69, 3.20]	3.67 (1.57)	[3.52, 3.82]
Eudaimonic Experiences	4.27 (1.40)	[4.00, 4.54]	3.50 (1.53)	[3.19, 3.80]	3.63 (1.29)	[3.37, 3.89]	2.94 (1.20)	[2.71, 3.17]	3.58 (1.44)	[3.44, 3.72]
ToM	4.18 (1.14)	[3.96, 4.40]	4.28 (1.06)	[4.07, 4.49]	4.40 (1.01)	[4.20, 4.60]	4.07 (1.12)	[3.85, 4.28]	4.23 (1.09)	[4.12, 4.33]
Emotion Recognition^b^	25.2 (4.32)	[24.4, 26.1]	24.9 (4.73)	[23.9, 25.9]	25.0 (4.72)	[24.1, 26.0]	25.4 (4.380	[24.6, 26.3]	25.2 (4.52)	[24.7, 25.6]
Study 2										
Emotional Experiences	4.74 (1.12)	[4.52, 4.96]	4.57 (1.34)	[4.31, 4.83]	3.77 (1.45)	[3.48, 4.05]	3.09 (1.26)	[2.84, 3.34]	4.04 (1.45)	[3.90, 4.19]
Eudaimonic Experiences	4.74 (1.01)	[4.54, 4.94]	3.70 (1.17)	[3.47, 3.93]	4.78 (1.38)	[4.50, 5.05]	3.43 (1.26)	[3.18, 3.68]	4.16 (1.35)	[4.03, 4.29]
ToM	4.19 (1.15)	[3.97, 4.42]	4.11 (1.15)	[3.88, 4.34]	4.38 (1.04)	[4.17, 4.58]	4.09 (1.05)	[3.88, 4.30]	4.19 (1.10)	[4.08, 4.30]
Emotion Recognition^b^	24.8 (4.75)	[23.8, 25.8]	24.7 (4.51)	[23.8, 25.6]	24.7 (5.44)	[23.6, 25.8]	24.4 (4.89)	[23.4, 25.4]	24.6 (4.89)	[24.1, 25.1]

*Note*. *N* = 401 in Study 1 and *N* = 395 in Study 2. SIP = Social Information Processing.

^a^Average descriptives across four conditions.

^b^*N* = 378 in Study 1 and *N* = 384 in Study 2 for Emotion recognition due to exclusion of participants who had technical problems.

#### SIP manipulation check

The manipulation check showed that the social information processing manipulation was successful. Participants in the deep social information processing condition (*M* = 5.94, *SD* = 1.18) reported greater involvement with the experience of the characters depicted in the images as compared to participants in the control condition (*M* = 3.91, *SD* = 1.71), *F* (3, 397) = 195.02, *p* <. 001, η_*p*_^2^ = .329. The effect of art (*M* = 5.25, *SD* = 1.66) vs. non-art (*M* = 4.60, *SD* = 1.86) was also significant and positive, albeit considerably smaller than the effect of social information processing, *F* (3, 397) = 16.58, *p* < .001, η_*p*_^2^ = .040. The interaction effect was not significant, *F* (3, 397) = 2.87, *p* = .091, η_*p*_^2^ = .007.

#### Emotional experience

**Fig 3:**
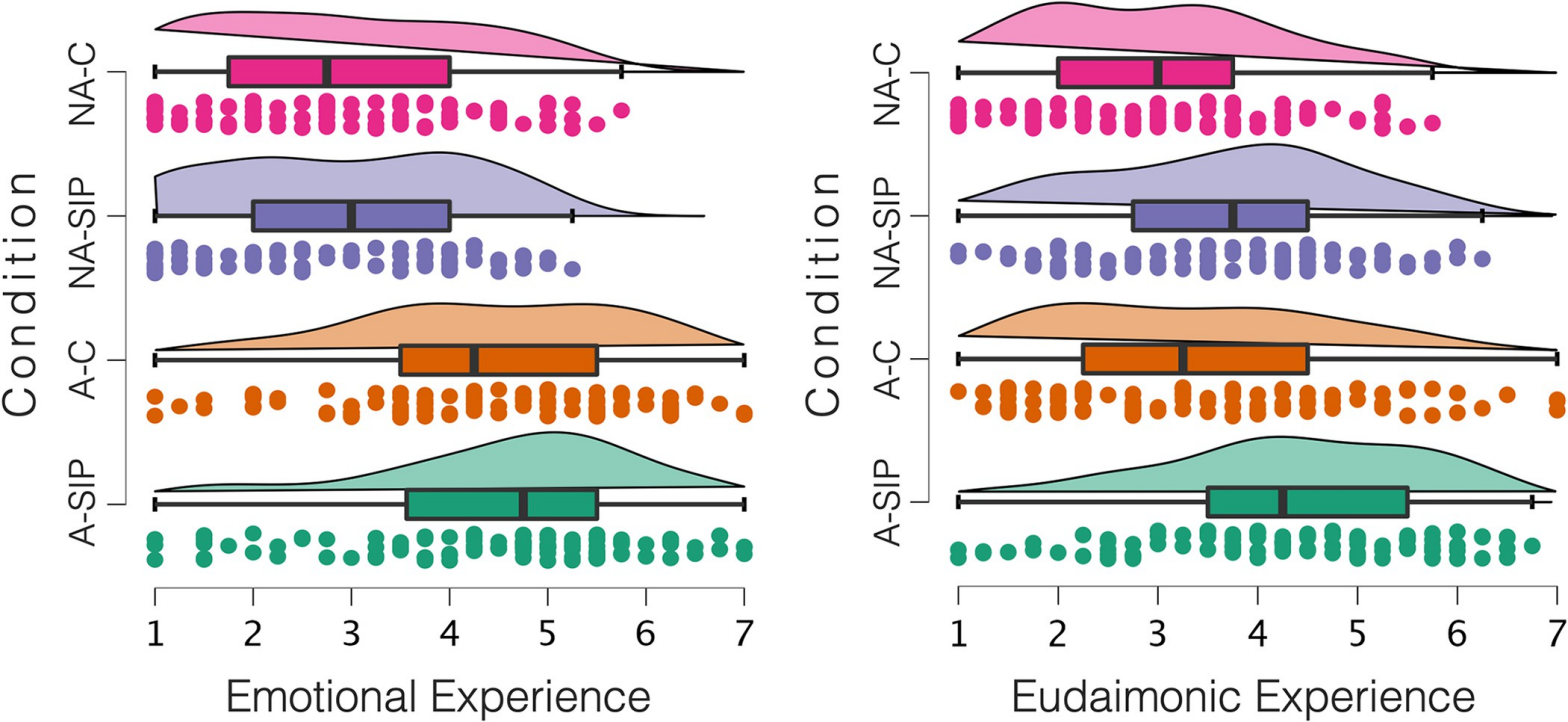
Emotional and eudaimonic experience as a function of art engagement and SIP in Study 1. Condition: A-SIP = Art–Deep Social Information Processing, A-C = Art–Control, NA-SIP = Non-art–Deep Social Information Processing, NA-C = Non-art–Control.

As hypothesized, engagement with art (*M* = 4.40, *SD* = 1.49) vs. non-art (*M* = 2.95, *SD* = 1.29) resulted in heightened emotional experience, *F*(3, 397) = 108.34, *p* < .001, η_p_^2^ = .214, while social information processing did not have a significant effect, *F*(3, 397) = 0.23, *p* = .628, η_p_^2^ = .001. The interaction effect between art and social information processing was not significant, *F*(3, 397) = 0.13, *p* = .716, η_p_^2^ < .001. Results support our hypothesis that art engagement evokes heightened emotional experiences. Results are illustrated in Fig 3.

**Eudaimonic experience.** Art engagement resulted in increased eudaimonic experience (*M*_art_ = 3.89, *SD*_art_ = 1.51; *M*_non-art_ = 3.27, *SD*_non-art_ = 1.29), *F*(3, 397) = 19.52, *p* < .001, η_p_^2^ = .047. The main effect of social information processing was also significant and positive (*M*_Deep_ = 3.96, *SD*_Deep_ = 1.38; *M*_Control_ = 3.21, *SD*_Control_ = 1.39), *F*(3, 397) = 29.11, *p* < .001, η_p_^2^ = .068. The interaction effect was not significant, *F*(3, 397) = 0.10, *p* = .756 η_p_^2^ < .001. These results support our hypothesis that art engagement increases eudaimonic experience, and show that deep social information processing also strengthens eudaimonic experience. Results are depicted in Fig 3.

#### Theory of mind

Scores for the false-belief task did not differ between the art and non-art conditions, *F*(3, 397) = 0.002, *p* = .962, η_p_^2^ < .001, nor between levels of social information processing, *F*(3, 397) = 1.12, *p* = .291, η_p_^2^ = .003. The interaction effect was marginally significant, *F*(3, 397) = 3.99, *p* = .046, η_p_^2^ = .010. However, probing the interaction showed that none of the pairwise comparisons were significant after Bonferroni correction. Results suggest that neither art nor its interaction with social information processing influenced ToM. Controlling for memory in ToM assessment did not change these results.

#### Emotion recognition

Twenty-three participants reported having technical problems while displaying GERT-S videos and were excluded from the analysis. There was no difference on the scores across conditions of art engagement, *F*(3, 374) = 0.08, *p* = .775, η_p_^2^ < .001, or conditions of social information processing, *F*(3, 374) = 0.003, *p* = .957, η_p_^2^ < .001. The interaction effect was not significant, *F*(3, 374) = 0.59, *p* = .442, η_p_^2^ = .002. Thus, neither art engagement nor its interaction with social information processing influenced emotion recognition skill.

### Discussion

Our findings showed that engaging with artworks evoked stronger emotional and eudaimonic experiences than engaging with non-art images, supporting our hypothesis with regards to personal aesthetic experience. In addition, social information processing increased eudaimonic experiences, aligning with literature connecting eudaimonic experiences to a deeper understanding of others’ psychology and social reality [21, 22].

We did not find an effect of art engagement on ToM or emotion recognition skill, nor did we find an additive effect resulting from the combination of art engagement and deep social information processing.

## Study 2

Having demonstrated the effect of art on personal aesthetic experience, and failing to find an effect on social cognitive skills, in Study 2 we aimed to (1) replicate these findings, (2) gain additional insight into the null effects on social cognitive skills by running the study under confirmatory hypotheses predicting a null effect, and (3) strengthen the social information processing manipulation for a second trial. Specifically, we hypothesized that art evokes intensified emotional and eudaimonic experiences, whereas it does not affect social cognitive skills of ToM and emotion recognition. Additionally, we employed Bayesian analyses to gain further insight into the hypothesized null effects in Study 2. The preregistration is available at the Open Science Framework: https://osf.io/va74f.

In Study 2 we also adjusted our social information processing manipulation to corroborate the involvement of the process route during art engagement. First, we standardized the involvement of social information processing while engaging with the material by adding narrative text to the images. This way, instead of asking participants to come up with text that described the experience of the characters (i.e., Study 1), we provided this information to them. Such verbal cues have an important role in inference making during art engagement, as they guide the viewer in the interpretation of the simulated experience [57]. Further, rather than using 3 focal images we implemented narrative text for all images.

### Method

#### Participants

A priori power analysis demonstrated that 405 participants were needed to detect a small to medium effect size (*f* = 0.165) in a 2-way ANOVA with α = .05 and power of .80. The actual sample consisted of 399 participants, recruited through Prolific in May 2022, who participated online via Qualtrics in exchange for money. Four participants were removed from the analyses due to failing an attention check, leaving a final sample of 395 (49.5% female, *M*_age_ = 43.8, *SD*_age_ = 14.5). All participants gave written informed consent prior to the experiment. Data collection was anonymized, and the authors did not have access to any identifying information after the data collection. The experiment was approved by the Ethics Review Board of the University of Amsterdam (2022-SP-15245).

#### Design and procedure

Participants were randomly assigned to a 2 (art engagement: yes vs. no) x 2 (social information processing: deep vs. control) between-subjects experimental design. The manipulation of art engagement was the same and the stimuli comprised seven painting-photo pairs chosen from the set used in Study 1. For the manipulation of social information processing, we either asked participants to acknowledge the experience of the people depicted and included narrative text to accompany the images (deep), or presented the images without any social information processing instructions (control). Each narrative text was 6–8 sentences long and was written by the authors. They described aspects of the characters’ experience and contained information about their life stories. For example, the following narrative accompanied the painting *At Eternity’s Gate* by Van Gogh and its non-art counterpart in the deep social information processing condition:

*The notice from the bank about his debt was the final straw. Daniel was weary of financial troubles, and now he was losing his house. He thought of gambling again. First a thrill, and then shame, sharply filled his body. He slammed his hands on his face, to hide the shame and punish the thrill. Who was to see him in the empty room? He was all alone. He yearned for someone to share the trouble with. He yearned for someone to have been there to keep him out of the trouble. He just didn’t see any way moving forward.*

Following the experimental manipulation, we assessed personal aesthetic experience and social cognitive skills using the same measures as in Study 1.

#### Analysis strategy

In the following results section, we report Bayesian ANOVA results along with frequentist analyses concerning our preregistered null hypothesis on the effect of art on social cognitive skills (i.e., ToM and emotion recognition skill). This is because the Bayes factor, unlike the *p*-value, can state evidence for/against a null hypothesis through estimating the ratio of probabilities of respective hypotheses based on the data [58]. Accordingly, we will report BF_01_, the Bayes factor which quantifies the support for the null hypothesis over the alternative. Further details on the reported Bayesian analyses, as well as Bayesian ANOVA results for other dependent variables in Studies 1 and 2, can be found in the S1 File. The analyses were performed using Jamovi [59, 60].

### Results

Descriptive statistics for the manipulation check and main dependent variables are presented per condition in Table 1.

#### Emotional experience

**Fig 4:**
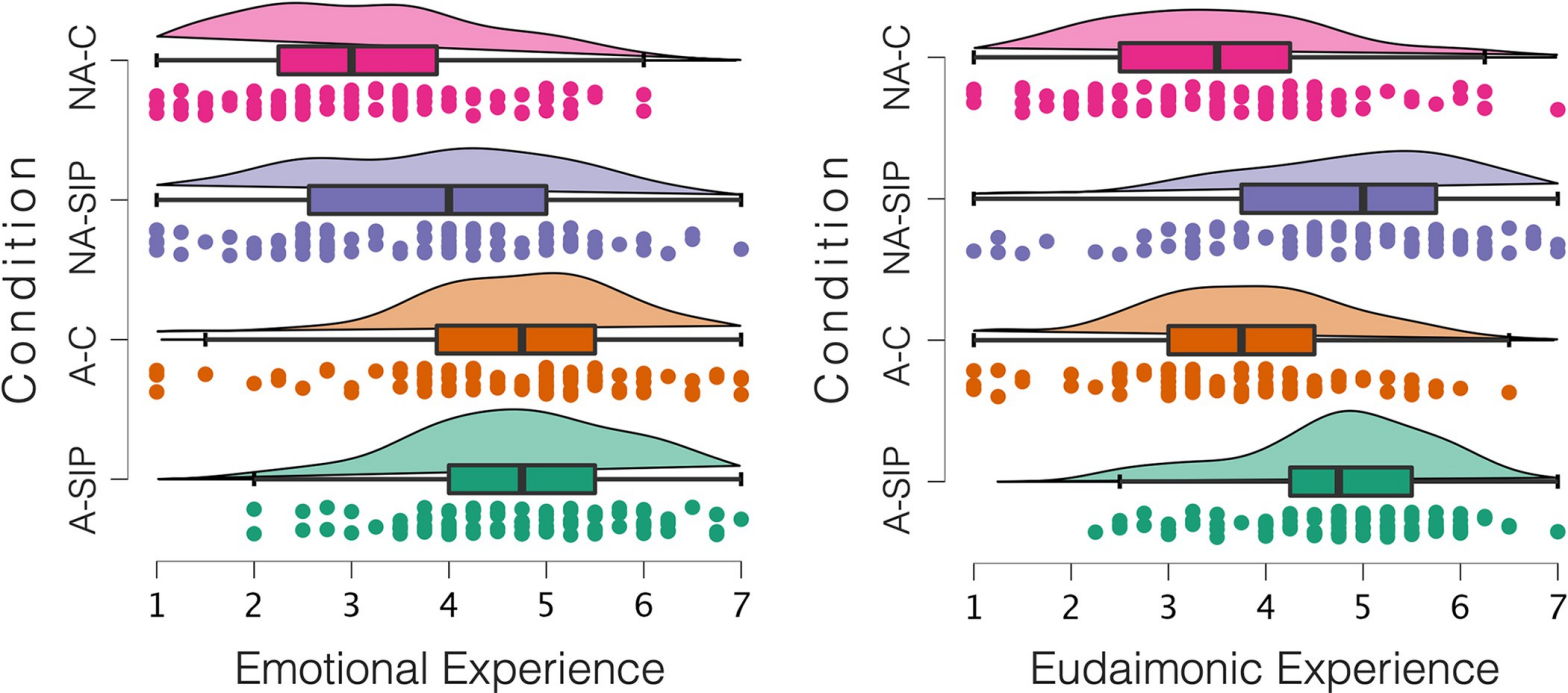
Emotional and eudaimonic experience as a function of art engagement and SIP in Study 2. Condition: A-SIP = Art–Deep Social Information Processing, A-C = Art–Control, NA-SIP = Non-art–Deep Social Information Processing, NA-C = Non-art–Control.

As hypothesized, art engagement resulted in heightened emotional experience (*M*_art_ = 4.66, *SD*_art_ = 1.23; *M*_non-art_ = 3.43, *SD*_non-art_ = 1.40), *F*(3, 391) = 88.28, *p* < .001, η_p_^2^ = .18. The effect of social information processing was also significant and positive (*M*_Deep_ = 4.26, *SD*_Deep_ = 1.38; *M*_Control_ = 3.83, *SD*_Control_ = 1.50), *F*(3, 391) = 10.47, *p* < .001, η_p_^2^ = .026. The interaction effect was not significant, *F*(3, 391) = 3.62, *p* = .058, η_p_^2^ = .009. Results are illustrated in Fig 4.

#### Eudaimonic experience

Art engagement did not influence eudaimonic experiences, *F*(3, 391) = .89, *p* = .347, η_p_^2^ = .002, while the main effect of social information processing was significant and positive, (*M*_Deep_ = 4.76, *SD*_Deep_ = 1.21; *M*_Control_ = 3.56, *SD*_Control_ = 1.22), *F*(3, 391) = 95.901, *p* < .001, η_p_^2^ = .20. The interaction effect was not significant, *F*(3, 391) = 1.52, *p* = .218, η_p_^2^ = .004. This does not support our hypothesis that art engagement enhances eudaimonic experiences. Nevertheless, this result replicates the finding of Study 1, where deep social information processing promotes eudaimonic experiences. Results are illustrated in Fig 4.

#### Theory of mind

Art engagement did not influence ToM performance, *F*(3, 391) = .56, *p* = .455, η_p_^2^ = .001. The effect of social information processing was also not significant, *F*(3, 391) = 2.76, *p* = .097, η_p_^2^ = .007. The interaction effect was not significant, *F*(3, 391) = .87, *p* = .353, η_p_^2^ = .002.

Looking at the Bayesian ANOVA results comparing four alternative models including (1) the main effect of art engagement, (2) the main effect of social information processing, (3) both main effects, and (4) both main effects and the interaction against the null model, we see that our data is almost 7 times more likely to occur under the null hypothesis compared to under the model with the main effect of art engagement (BF_01_ = 6.85). Further, the data is 69 times more likely to occur under the null hypothesis than under the model with both main effects and the interaction (BF_01_ = 69.35). In other words, there is moderate to very strong evidence supporting the null hypothesis where neither art engagement, nor its combination with social information processing influences ToM performance, which is in line with our preregistered hypothesis.

#### Emotion recognition

Eleven participants reported having technical problems while watching GERT-S videos, and were excluded from the analysis. There was no difference on the scores across conditions of art engagement, *F*(3, 380) = 0.15, *p* = .696, η_p_^2^ < .001, or social information processing, *F*(3, 380) = 0.18, *p* = .675, η_p_^2^ < .001. The interaction effect was not significant, *F*(3, 380) = 0.03, *p* = .872, η_p_^2^ < .001.

Bayesian ANOVA results also yielded a congruent picture. Comparing the null model with the four alternative models including (1) the main effect of art engagement, (2) the main effect of social information processing, (3) both main effects, and (4) both main effects and the interaction; our data was 8 times more likely to occur under the null hypothesis than the model with the main effect of art (BF_01_ = 8.24), and 422 times more likely to occur under the null hypothesis compared to the model with the two main effects and the interaction (BF_01_ = 422.8). The Bayes factors yield moderate to overwhelming evidence supporting the null hypothesis, and results converge on the conclusion that neither art nor its interaction with social information processing facilitate emotion recognition skill, which is in line with our preregistered hypothesis.

### Discussion

In Study 2, consistent with Study 1 findings, engaging with artworks intensified participants’ emotional experience as compared to engaging with non-artistic images. However, contrary to our hypothesis and previous findings, art engagement did not facilitate eudaimonic experiences. On the other hand, deep social information processing facilitated both eudaimonic and emotional experiences.

Our preregistered expectations on the null findings concerning performance on social cognitive skills were supported. Art engagement facilitated neither ToM nor emotion recognition skills. Social information processing also did not influence either social cognitive skill.

## General discussion

In the current study, we investigated the effect of art engagement on personal aesthetic experience, and tested whether engaging with visual art can affect individuals beyond such emotional and eudaimonic experiences, specifically, fostering social cognitive skills.

Literature has shown that art engagement evokes a wide range of emotional and eudaimonic outcomes [e.g., 4]. The majority of previous findings are based on research that examined engagement with artworks, lacking an effective control condition of non-art stimuli. Other studies that did include a non-art control assessed the effect of framing (i.e., art versus non-art), essentially testing art schema effects. Here we examined art engagement using veridical artworks and experimentally matched non-art photos, ensuring the ecological validity of art engagement and a comparison with an effective control condition. Across two studies we found that art engagement intensified emotional experiences as measured by prototypical aesthetic emotions. However, our findings on eudaimonic experiences were inconsistent. Specifically, while art facilitated eudaimonic experiences in Study 1, we failed to show this effect in Study 2. Results suggest that the discrepancy stems from heightened eudaimonic experience following non-art engagement in Study 2, which may signal a research participation effect [61] where participants took an inquisitive stance to the non-art photos presented without any instructions. On the other hand, deep social information processing consistently heightened eudaimonic experiences in both studies. This is in line with literature connecting eudaimonic experiences to a deeper understanding of the human condition, facilitated by taking a perspective on others’ social reality [21, 22]. While previously this relationship was constructed by suggesting that eudaimonia, or a pursuit of meaning and insight, increased awareness of other’s experiences [19], we showed the effect in the opposite direction, demonstrating heightened eudaimonic experience as a consequence of deep social information processing.

Across studies, we did not find any effect of engagement with paintings on social cognitive skills, measured as either ToM or emotion recognition. Taken together with findings from literary fiction and theatre [e.g., 7], our results highlight the nuanced effect of art engagement, calling attention to the mechanism present in engagement with those artforms, that engagement with paintings may lack. Rathje and colleagues [62], while contrasting literary fiction and theatre to other artforms, highlighted that these are the prominent forms of narrative art, centred around character development and human stories, making them more likely to engender stronger interpersonal outcomes. Compared to these narrative forms, paintings present a static, “frozen” representation of the moment, rather than the dynamic flow of a fictional story. It is, nevertheless, possible to “read” the story of a character from a painting through their facial expression and posture, or by pondering their thoughts, emotions, and intentions, like we did with our social information processing manipulation. Nevertheless, this reading is rather indirect and self-led compared to the flow of prose fiction, and guidance of literary devices or genre conventions. Through its dynamic flow, it is likely that prose fiction, compared to paintings, more so allows the type of social cognition practice we laid out in our theoretical background, which could boost social cognitive skills.

Furthermore, understanding a character or simulating others’ experiences through a plot takes time and requires *enduring* engagement, whereas studies indicate that visitors, on average, spend under 25 minutes at an exhibition [63], and viewing times for a single artwork typically does not exceed 30s [41]. Indeed, research that showed the effect on literary fiction and theatre used longer engagement with a single work of art, such as an entire book [39] or the full theatre play [11]. Compared to these, the duration of art engagement in our studies were 2–4 minutes on average, and this involved viewing a set of artworks that depicted different characters and events. Thus, it may have been the case that the different artworks we presented did not allow for a unified, enduring engagement with a certain character, eliciting emotions congruent to their experience as it unfolds, which would otherwise be allowed by the narrative of literary fiction or a theatre play.

Moreover, we noted that artistic depictions that are to achieve this effect involve an optimal amount of ambiguity that prompts viewers (or readers) to fill the gaps by making inferences and putting themselves in the shoes of the characters [24, 36]. We argued that ambiguity inherent in paintings could be a vehicle for the effect. However, it is possible that a static scene depicted in an artwork may be too ambiguous in terms of the information available to “complete” character stories, or on the contrary, visual cues may be too explicit that there are not any holes left to fill by imagination or imagery. Mar and Oatley [24] emphasized that imagery evoked by literature is a central part of the simulation of social experiences as it could allow the reader, differently from visual arts, to engage with the subjective experiences of the characters. Similarly, Lodge [64], while explaining the tie between prose and imagination, said ‘‘visual arts, can do more immediate justice to the visible world, but cannot match the power of language to mean” (p. 153). Perhaps in this case, a picture was indeed worth a thousand words, so that it left little to the imagination.

Arguably, there are limitations posed by the procedural choices made in our study design. Namely, our studies were online experiments. It could be argued that computer-mediated visual art engagement may differ from an art encounter with the physical, original artwork. However, research that contrasted digital reproductions to the original artwork showed that aesthetic evaluations did not differ between the two forms [65], and viewers focused on the artistic aspects unbound by the medium of presentation [66]. Our studies featured art and non-art images sourced from the internet, presented in a format resembling how people commonly interact with images online. Thus, we believe that our results are reproducible and would generalize to other samples online with different forms of visual art engagement. This way, our findings suggest that emotional and eudaimonic experiences are remarkably accessible to a broad audience; anyone with internet access could benefit from evoking these experiences [e.g., 2, 5, 19, 20] by briefly browsing art online. Nevertheless, future research could shed light on the implications of *offline* visual art engagement (e.g., at the museum) on social cognitive skills. Further, we acknowledge that social information processing is a complex, multidimensional construct, which is involved in all forms of social interaction within various levels of complexity (e.g., domain and modality of social information). Future research could employ alternative operationalizations that tap into different aspects of this construct.

In the current studies, we showed that art encounters alter personal aesthetic experience through changes in emotional and eudaimonic outcomes. On the other hand, we did not find any support for the effect of art engagement beyond changes in personal experience. All in all, our investigation has expanded the literature on the effects of art engagement on social cognition by testing whether the effects found for engaging with literary fiction and theatre generalized to engagement with paintings. While we did not find any evidence of such generalizability, we emphasize that various forms of art may differ in terms of what they offer to the viewer. This adds to a more nuanced understanding of the effect of art engagement, both across different domains of art, and across measuring formats of the outcome variables. Literature lumped self-report and performance-based measures together under social cognitive skills [e.g., 7], even though these may assess different aspects of a construct (i.e., individual’s perception of their ability vs. objective performance). Research that tested both self-report and performance-based measures of social cognition highlighted the discrepancy between the two formats, such as the absence of any correlation [67]. Performance-based ability measures like the GERT-S employed here are developed to measure individual differences, and differences across cultures or clinical and non-clinical populations [51, 56]. They are apt to assess stable, trait-like abilities. For instance, Fernández-Abascal and colleagues [68] showed that RMET [51] is a reliable measure of ToM that shows stability over a 1-year period. On the contrary, it is not clear whether these measures are apt to measure, or appropriate to use for measuring, immediate or momentary differences that could follow a brief experience. Indeed, some research showed the effect on RMET [10] while others failed to replicate it [e.g., 9]. Future work should clarify what this difference between the two formats means for the effect of art and its conceptualization. It is possible that a change in performance on social cognitive skills is only plausible after long-term, recurring engagement.

As we have seen that the effect of art engagement is nuanced, the practical implications should also follow. When including art interventions in practical contexts such as education, we need to specify what we expect from the intervention and shape such curricula accordingly. The specific domain of art, artwork content, ways of engagement, duration, and frequency are all aspects that require further investigation and should shape what we expect to gain from art interventions.

## Supporting information

S1 File Supplementary material. Presents the full list of measures used in our studies and additional analyses not included in the manuscript.
(PDF)
