# Supplementary material for: Beyond beauty: Does visual art facilitate social cognitive skills?
Source: PLoS One. 2024 Oct 4;19(10):e0308392. doi: 10.1371/journal.pone.0308392 (PMC11452045; doi:10.1371/journal.pone.0308392)
Supplement: S1 File — Presents the full list of measures used in our studies and additional analyses not included in the manuscript. (PDF) [file pone.0308392.s001.pdf]

Supplementary Material

Here we provide supplementary methodological and statistical information for the studies reported in the main text. We first explain the pilot study conducted for stimuli selection and validation in Section 1. Then, we report the results not included in the main text in Sections 2-4. Under Section 2, we report analyses on control variables that eliminated possible confounds. Under Section 3, we report Bayesian ANOVA tables for the focal outcome variables, followed by intercorrelations among focal outcome variables in Section 4. We then report exploratory analyses on non-focal outcome variables under Section 5. Finally, we provide the list of scales, and the ToM task used in Studies 1 and 2 under Supplementary Appendix A, followed by narrative texts used for social information processing manipulation in Study 2. Data is available on Open Science Framework: https://osf.io/akd9u/.

Section 1: Pilot Study

The goal of the pilot study was to select and validate the experimental stimuli we used in Studies 1 and 2. Our aim was to statistically match the art and non-art images in terms of valence and viewers' identification with the depicted characters. Identification was matched to ensure that participants were equally inclined to engage in social information processing regarding the characters in paintings and photos, as this would influence our manipulation of social information processing. Last but not least, we tested whether the art images were considered more artistic than their matched non-art images.

Method

Participants

Seventy-eight undergraduate students from the University of Amsterdam participated in the study through the university's subject pool in March 2022. The study was conducted in English. Participants completed the study online via an anonymized Qualtrics survey and

were rewarded with research credit[1]. All participants gave written informed consent prior to the study. The study was approved by the Ethics Review Board of the University of Amsterdam (2022-SP-14608).

Design and Procedure

We collected painting and photo pairs, which were either found online or taken from submissions to the Getty Museum Challenge. This challenge started when the Getty Museum invited its followers on Twitter to recreate a work of art as a photo with objects in their homes (see twitter.com/GettyMuseum/status/1242845952974544896). We compiled 40 pairs of images that visually matched in terms of content, colors, and composition (80 images in total). All pairs depicted social actors to stimulate the content route (Oatley, 2016). The images used in the Pilot Test are available upon request from the authors.

We tested the image pairs on artfulness, emotional valence, and social identification with individuals depicted. Participants were randomly assigned to view either 20 paintings or 20 photographs out of 40 images in each category. They viewed the images one by one and rated each image on how artistic they found it (artfulness), how much they identified with the person depicted in the image (identification), and how positive or negative the emotion conveyed by the image was (valence). In addition, we assessed perceived artistic impact of the painting set by asking participants to what extent they viewed each painting as an impactful work of art. All questions were answered on 7-point Likert scales, with the valence question ranging from “very negative” to “very positive” and others from “not at all” to “very much”.

Results

Each pair was tested with independent t-tests on artfulness, valence, and social identification. We selected pairs that did not differ in valence and identification, and those

[1] Demographics for the Pilot Study were not recorded due to a technical error.

that differed in artfulness. Thirteen pairs met these criteria. Out of these pairs, we selected 9 paintings with the highest scores on artistic impact. Mean ratings for the focal variables are displayed in Supplementary Fig 1. Additionally, we compared the final art and non-art sets with independent t-tests on artfulness, valence, and social identification. The two sets differed in artfulness, $t(16) = 9.29$, $p < .001$, $d = 4.38$, CI [2.07, 6.67], as artworks were perceived significantly more artistic, while they did not differ in valence, $t(16) = -0.35$, $p = .730$, $d = -.17$, 95% CI [-1.09, 0.77], or social identification, $t(16) = 0.62$, $p = .543$, $d = .29$, CI [0.29, -0.65].

Supplementary Fig 1

Descriptives for Image Pairs Selected in the Pilot

Pair	Artist, Title, and Year	Art Condition			Non-Art Condition		
		Artfulness *M* (*SD*)	Valence *M* (*SD*)	Identification *M* (*SD*)	Artfulness *M* (*SD*)	Valence *M* (*SD*)	Identification *M* (*SD*)
1	Pierre-Auguste Renoir, *Le Déjeuner des canotiers*, 1881	5.36 (1.08)	5.86 (0.95)	3.71 (1.64)	4.17 (1.56)	5.78 (0.95)	3.17 (1.53)
2	Vincent van Gogh, *At Eternity's Gate,* 1890	5.63 (1.06)	1.84 (1.54)	3.53 (1.84)	2.91 (1.51)	2.27 (1.28)	3.36 (1.36)
3	Frederic Leighton, *Flaming June*, 1895	6.00 (1.18)	4.86 (0.86)	4.86 (1.46)	3.43 (1.78)	4.39 (1.23)	4.13 (1.46)
4	Pablo Picasso, *Woman Ironing*, 1904	6.00 (0.96)	2.36 (1.50)	2.64 (1.39)	3.91 (1.47)	2.78 (0.85)	3.09 (1.31)
5	Gustave Courbet, *The Desperate Man*, 1844–1845	6.05 (1.13)	2.74 (1.52)	3.16 (1.50)	4.27 (1.35)	2.91 (0.97)	2.91 (1.54)
6	Gustav Klimt, *The Kiss*, 1907-1908	6.00 (1.18)	4.86 (0.86)	4.86 (1.46)	3.43 (1.78)	4.39 (1.23)	4.13 (1.46)
7	Pablo Picasso, *The Absinthe Drinker*, 1901	5.26 (1.24)	3.32 (1.20)	3.53 (1.47)	3.36 (1.14)	3.82 (0.85)	3.14 (1.36)
8	George Hendrik Breitner, *Girl in a White Kimono*, 1894	5.57 (1.28)	3.00 (1.71)	3.50 (2.03)	3.96 (1.87)	3.04 (1.30)	3.17 (1.64)
9	Jean-François Millet, *The Man with the Hoe,* 1860-1862	5.50 (1.02)	2.64 (1.65)	1.64 (0.74)	2.43 (1.31)	3.87 (0.63)	2.13 (1.42)

Section 2: Analyses pertaining to Control Variables

Manipulation Check on Experimental Task Difficulty for Study 1

In Study 1, we assessed task difficulty regarding our social information processing manipulation, because writing about another's experience may require more effort than a simple task of listing colors and objects one sees. After the experimental manipulation we asked participants to rate the experimental task on difficulty on a 10-point Likert-scale.

We performed an ANOVA to compare difficulty across experimental conditions. Results showed that participants in the deep social information processing condition (M = 3.79, SD = 2.36) rated the task as more difficult $F(3, 397) = 38.20$, p <.001, $\eta_p^2 = .088$, compared to the participants in the control condition (M = 2.46 , SD = 1.89). Art also significantly and positively influenced the ratings $F(3, 397) = 5.19$, p =.023 , η_p^2 =.013. The interaction effect was not significant $F(3, 397) = 1.01$, p =.315 , η_p^2 =.003. Because participants rated the task to be more difficult in both art and social information processing conditions, we included difficulty as a covariate in the analyses on the focal outcome variables. However, controlling for difficulty did not change the size or direction of the effects on any of our focal outcome variables pertaining to personal aesthetic experience (i.e., prototypical aesthetic emotions and eudaimonic experience) or social cognitive skills (i.e., Theory of Mind (ToM) and emotion recognition).

Controlling for Art interest in Studies 1 and 2

Art interest was added as a covariate in the analyses of experimental manipulation on focal outcome variables pertaining to personal aesthetic experience and social cognitive skills. Controlling for art interest did not change the size or direction of the effects on any of our focal outcome variables in either of the studies.

In addition, in order to explore associations between art interest and the outcome variables in both studies, we also conducted correlational analyses. In Study 1, art interest was positively associated with emotional experience $r = .29$, $p < .001$ and eudaimonic experience $r = 0.31$, $p < .001$. In Study 2, art interest again positively correlated with emotional experience $r = .27$, $p <.001$ and eudaimonic experience $r = .27$, $p <.001$, as well as emotion recognition $r = .12$, $p=.020$.

Section 3: Bayesian ANOVA tables for Outcome Variables

Study 1

Supplementary Table 1

Bayesian Anova Results for Emotional Experience in Study 1

Model Comparison - Emotional Experience

Models	P(M)	P(M|data)	BF_M	BF_{10}	% error
Null model	0.200	2.44e-20	9.78e-20	1.00	
Art Engagement	0.200	0.88	28.86	3.59e+19	1.24e-26
Social Information Processing	0.200	3.38e-21	1.35e-20	0.14	0.12
Art Engagement + Social Information Processing	0.200	0.10	0.46	4.24e+18	1.30
Art Engagement + Social Information Processing + Interaction	0.200	0.02	0.07	7.44e+17	1.43

Analysis of Effects

Effects	P(incl)	P(incl|data)	$BF_{Inclusion}$
Art Engagement	0.600	1.00	∞
Social Information Processing	0.600	0.12	0.09
Interaction	0.200	0.02	0.07

Note. N=401. BF_{10} is the Bayes factor informative on the likelihood of the alternative over the null hypothesis. $BF_{Inclusion}$, the Bayesian inclusion factor, is informative on whether the data supports the inclusion of an effect in a model. Here, the Bayes factor indicates that our data is 3.59e+19 times more likely to occur under the model including the main effect of art, supporting our hypothesis that art engagement evokes heightened emotional experiences.

Supplementary Table 2

Bayesian Anova Results for Eudaimonic Experience in Study 1

Model Comparison - Eudaimonic Experience

Models	P(M)	P(M|data)	BF_M	BF_{10}	% error
Null model	0.200	7.82e-9	3.13e-8	1.00	
Art Engagement	0.200	8.97e-6	3.59e-5	1146.84	2.54e-5
Social Information Processing	0.200	7.45e-4	0.00	95222.98	2.29e-11

Art Engagement + Social Information Processing	0.200	0.87	26.03	1.11e+8	2.93
Art Engagement + Social Information Processing + Interaction	0.200	0.13	0.61	1.69e+7	1.58

Analysis of Effects

Effects	P(incl)	P(incl|data)	$BF_{Inclusion}$
Art Engagement	0.600	1.00	894.26
Social Information Processing	0.600	1.00	74241.64
Interaction	0.200	0.13	0.611

Note. N=401. The Bayes factor indicated overwhelming evidence in favor of the alternative hypothesis for the model including two main effects (BF_{10} = 1.11e+8), suggesting both art engagement and social information processing heightens eudaimonic experience.

Supplementary Table 3

Bayesian Anova Results for Theory of Mind in Study 1

Model Comparison - ToM

Models	P(M)	P(M|data)	BF_M	BF_{01}	% error
Null model	0.200	0.74	11.59	1.00	
Art Engagement	0.200	0.08	0.36	9.04	0.14
Social Information Processing	0.200	0.14	0.66	5.22	0.09
Art Engagement + Social Information Processing	0.200	0.02	0.06	46.71	1.15
Art Engagement + Social Information Processing + Interaction	0.200	0.02	0.06	46.71	1.50

Analysis of Effects

Effects	P(incl)	P(incl|data)	$BF_{Inclusion}$
Art Engagement	0.600	0.11	0.09
Social Information Processing	0.600	0.17	0.14
Interaction	0.200	0.02	0.06

Note.N=401. BF_{01} is informative on the likelihood of the null over the alternative hypothesis. The Bayes factor supports the null model over the alternatives. For instance, our data is 46.71 times more likely to occur under the null hypothesis compared to the model with two main effects and the interaction. This suggests that neither art nor its interaction with social information processing influenced ToM.

Supplementary Table 4

Bayesian Anova Results for Emotion Recognition in Study 1

Model Comparison – Emotion Recognition

Models	P(M)	P(M|data)	BF_M	BF_{01}	% error
Null model	0.200	0.80	16.05	1.00	
Art Engagement	0.200	0.09	0.42	8.41	0.13
Social Information Processing	0.200	0.09	0.40	8.76	0.13
Art Engagement + Social Information Processing	0.200	0.01	0.04	76.03	1.17

Art Engagement + Social Information Processing + Interaction	0.200	0.00	0.01	330.94	7.38

Analysis of Effects

Effects	P(incl)	P(incl|data)	$BF_{Inclusion}$
Art Engagement	0.600	0.11	0.08
Social Information Processing	0.600	0.10	0.08
Interaction	0.200	0.00	0.01

Note. N=378. The Bayes factor indicated very strong evidence in favor of the null hypothesis over the model with two main effects and the interaction (BF_{01} = 330.94), suggesting that neither art nor the interaction with social information processing influenced participants' emotion recognition abilities.

Study 2

Supplementary Table 5

Bayesian Anova Results for Emotional Experience in Study 2

Model Comparison - Emotional Experience

Models	P(M)	P(M|data)	BF_M	BF_{10}	% error
Null model	0.200	9.76e-18	3.91e-17	1.00	
Art Engagement	0.200	0.03	0.14	3.41e+15	2.18e-22
Social Information Processing	0.200	6.73e-17	2.69e-16	6.89	0.00
Art Engagement + Social Information Processing	0.200	0.53	4.46	5.40e+16	0.81
Art Engagement + Social Information Processing + Interaction	0.200	0.44	3.13	4.50e+16	1.15

Analysis of Effects

Effects	P(incl)	P(incl|data)	$BF_{Inclusion}$
Art Engagement	0.600	1.00	6.00e+15
Social Information Processing	0.600	0.97	19.33
Interaction	0.200	0.44	3.13

Note. N=395. The Bayes factor indicated overwhelming evidence in favor of the alternative hypothesis for the model including the two main effects (BF_{10} = 5.40e+16).

Supplementary Table 6

Bayesian Anova Results for Eudaimonic Experience in Study 2

Model Comparison - Eudaimonic Experience

Models	P(M)	P(M|data)	BF_M	BF_{10}	% error
Null model	0.200	4.59e-18	1.84e-17	1.00	
Art Engagement	0.200	7.37e-19	2.95e-18	0.16	0.10

Social Information Processing	0.200	0.82	18.48	1.79e+17	3.30e-24
Art Engagement + Social Information Processing	0.200	0.14	0.63	2.96e+16	1.45
Art Engagement + Social Information Processing + Interaction	0.200	0.04	0.18	9.19e+15	2.19

Analysis of Effects

Effects	P(incl)	P(incl|data)	$BF_{Inclusion}$
Art Engagement	0.600	0.18	0.14
Social Information Processing	0.600	1.00	infinite
Interaction	0.200	0.04	0.18

Note. N=395. The Bayes factor indicated overwhelming evidence in favor of the alternative hypothesis over the null hypothesis for the model including the main effect of social information processing (BF_{10} = 1.79e+17), as well as the model including two main effects (BF_{10} = 2.96e+16).

Supplementary Table 7

Bayesian Anova Results for Theory of Mind in Study 2

Model Comparison - ToM

Models	P(M)	P(M|data)	BF_M	BF_{01}	% error
Null model	0.200	0.61	6.30	1.00	
Art Engagement	0.200	0.09	0.39	6.89	0.11
Social Information Processing	0.200	0.26	1.38	2.39	0.043
Art Engagement + Social Information Processing	0.200	0.03	0.15	17.27	1.41
Art Engagement + Social Information Processing + Interaction	0.200	0.01	0.03	74.38	2.23

Analysis of Effects

Effects	P(incl)	P(incl|data)	$BF_{Inclusion}$
Art Engagement	0.600	0.13	0.10
Social Information Processing	0.600	0.30	0.28
Interaction	0.200	0.01	0.03

Note. N=395. Interpretation for the Bayes factor in light of our hypotheses can be found in the main text.

Supplementary Table 8

Bayesian Anova Results for Emotion Recognition in Study 2

Model Comparison – Emotio Recognition

Models	P(M)	P(M|data)	BF_M	BF_{01}	% error
Null model	0.200	0.79	15.33	1.00	
Art Engagement	0.200	0.10	0.43	8.24	0.13
Social Information Processing	0.200	0.10	0.43	8.16	0.13
Art Engagement + Social Information Processing	0.200	0.01	0.05	67.25	1.15

Art Engagement + Social Information Processing + Interaction	0.200	0.00	0.01	452.41	2.62

Analysis of Effects

Effects	P(incl)	P(incl|data)	$BF_{Inclusion}$
Art Engagement	0.600	0.12	0.08
Social Information Processing	0.600	0.11	0.08
Interaction	0.200	0.00	0.01

Note. N=384. Interpretation for the Bayes factor in light of our hypotheses can be found in the main text.

Section 4: Intercorrelations among Outcome Variables

Supplementary Table 9

Intercorrelations Among Focal Outcome Variables in Studies 1 and 2

Study 1				Study 2			
	Prototypical Aesthetic Emotions	Eudaimonic Experience	ToM		Prototypical Aesthetic Emotions	Eudaimonic Experience	ToM
Prototypical Aesthetic Emotions	-			Prototypical Aesthetic Emotions	-		
Eudaimonic Experiences	.64***	-		Eudaimonic Experiences	.74***	-	
ToM	-.039	-.002	-	ToM	-.164***	-.158**	-
Emotion Recognition[b]	-.029	-.021	.24***	Emotion Recognition[b]	.012	.004	.16**

Note. N= 401 in Study 1 and *N*=395 in Study 2, **p < .05, **p <.01, ***p <.001.*
[b]*N* = 378 in Study 1 and *N* =384 in Study 2 for Emotion recognition due to exclusion.

Section 5: Exploratory Analyses on Non-focal Outcome Variables

Assessing the effect of Art and Social Information Processing on Negative Emotions in Studies 1 and 2

Apart from assessing participants' prototypical aesthetic emotions and eudaimonic experiences in Studies 1 and 2, we also assessed negative emotions in response to art experience and social information processing. Negative emotions were measured via ratings on two statements after the experimental manipulation, "I felt concerned" and "I felt

unsettled", on a 7-point Likert-scale. In Study 1, experimental task difficulty predicted heightened negative emotions *b* = .10, $t(396) = 3.22$, $p = .001$ and changed the size of the effect of social information processing, thus was added to the model as a covariate. ANCOVA results showed a significant effect of social information processing $F(4, 396) = 10.27$, $p = .001$, $\eta_p^2 = .025$, controlling for effort, where participants in the deep social information processing condition (*M* = 2.88, *SD* = 1.42) reported increased negative emotions after the viewing experience compared to participants in the control condition (*M* = 2.27, *SD* = 1.39). The effect of art $F(4, 396) = 0.10$, $p = .753$, $\eta_p^2 < .001$ and the interaction effect was not significant $F(4, 396) = 2.81$, $p = .094$, $\eta_p^2 = .007$ controlling for effort.

In Study 2, ANOVA results showed a significant boosting effect of social information processing $F(3, 391) = 112.909$, $p < .001$, $\eta_p^2 = .24$ on negative emotions, as well as a significant buffering effect of art $F(3, 391) = 6.87$, $p = .009$, $\eta_p^2 = .017$. The interaction effect was non-significant $F(3, 391) = 0.41$, $p = .524$, $\eta_p^2 = .001$.

Assessing the effect of Art and Social Information Processing on Donation Behavior in Study 1

We explored the effect of the experimental manipulation on prosocial behavior operationalized as donation to charity. Logistic regression was used to analyze the relationship between art and social information processing on the probability of donating. Neither art OR=1.14, $p = .532$, nor social information processing, OR=.94, $p = .784$, was a significant predictor of donating. The interaction term was also non-significant, OR=.48, $p = .078$. The odds of donating to charity were not influenced by whether participants engaged with art (vs. non-art), nor whether they took a social information processing stance while doing so.

Assessing the effect of Art and Social Information Processing on State Empathy in Study 2

Apart from assessing performance-based social cognitive skills of ToM and emotion recognition, in Study 2 we included a self-report measure of state empathy. The measure was composed of 6 pictures depicting people experiencing negative or positive emotions, selected from the International Affective Picture System (IAPS; Lang et al., 1997) followed by two statements to assess emotional ("I felt the emotion of the main character") and cognitive empathy ("I understood the situation of the main character") for the main character in each picture. The statements were rated on a 9-point Likert scale. As stated in our preregistered hypothesis on https://osf.io/va74f, we expected art experiences to increase self-reported empathy. We conducted ANOVAs separately for emotional and cognitive empathy to test this hypothesis.

For emotional empathy, results revealed neither art $F(3, 391) = 0.35$, $p = .552$, $\eta_p^2 = .001$, nor social information processing $F(3, 391) = 3.06$, $p = .081$, $\eta_p^2 = .008$ influenced self-reported emotional empathy. The interaction effect was also non-significant $F(3, 391) = 2.35$, $p = .126$, $\eta_p^2 = .006$. Importantly, when we looked at emotional empathy scores, 75% of the ratings exceeded 6.1, while 25% exceeded 7.7 out of 9, suggesting a ceiling effect.

For cognitive empathy, results revealed that neither art $F(3, 391) = 0.005$, $p = .944$, $\eta_p^2 < .001$, nor social information processing $F(3, 391) = 0.06$, $p = .813$, $\eta_p^2 < .001$ influenced self-reported cognitive empathy. The interaction effect was also non-significant $F(3, 391) = 0.31$, $p = .578$, $\eta_p^2 = .001$. Importantly, similar to emotional empathy, cognitive empathy scores suggested a ceiling effect where 75% of the ratings exceeded 6.5, and 25% exceeded 8 out of 9.

Supplementary Appendix A

Supplementary Table 10

Scales used for Emotional and Eudaimonic Outcomes, Negative Emotions and Art Interest in Studies 1 and 2

Scale	Items	Anchors
Prototypical Aesthetic Emotions	I felt a sense of beauty<br>I felt deeply moved<br>I felt awe<br>I felt fascinated	1 = not at all, 7 = very much (idem for all items)
Reflective Thoughts Scale	The images were thought-provoking<br>The images made me think about myself<br>The images inspired me to think about important issues<br>The images helped me to better understand other people	1 = not at all, 7 = very much (idem for all items)
Negative Emotions	I felt concerned<br>I felt unsettled	1 = not at all, 7 = very much (idem for all items)
Art Interest	I am interested in art	1 = not at all, 7 = very much

False-Belief Task Materials

ToM Story

Richard and Ann want to redecorate their spare bedroom. They have several wallpaper samples which they have brought home from the DIY shop. After choosing one, they decide to start the next weekend. Ann says she can collect the wallpaper on her way home from work next Friday evening. In the meantime, Richard keeps the sample wallpaper in the spare bedroom. On Thursday evening Ann puts the chosen sample wallpaper in the

garage while Richard is on a jog. She plans to take it to the DIY shop the next day to easily find the match. However, on Friday morning, she forgets it in the garage and leaves for work.

On Friday morning Richard's boss unexpectedly tells him he can take the afternoon off. When he gets home he phones Ann at work. Unfortunately she is not available. One of Ann's colleagues tells him she is at a meeting and she is not sure when Ann will be back. Richard explains that he wants to tell her he has got the afternoon off and he is going to start their redecorating. He says `Can you ask Ann to call me at home when she returns?' By the time Ann gets back to her office it is after 5 p.m. and all her colleagues have gone home. As she collects her things she sees a note on her desk saying that her husband called to tell her he had the afternoon off and was beginning their redecorating work. It is late and Ann wants to get to the DIY shop before 6 p.m. so she rushes down to buy the wallpaper.

At home Richard has been working very hard. He hasn't heard from Ann and he wonders why her colleagues are so unreliable. He's nearly finished stripping off the old wallpaper when he hears Ann coming through the front door. He rushes downstairs and says `Look what I've been doing this afternoon!'.

True/False Statements

(Correct answer provided in parenthesis following each statement; F = false, T = true)

1. Richard assumed Ann had received his message when she got home. (F)
2. Richard and Ann wanted to redecorate their spare bedroom. (Memory Statement, T)
3. While driving home Ann thought that Richard thought that her colleagues were unreliable and forgot to relay his message. (F)
4. Richard thinks Ann is going to be surprised to see his progress on redecorating. (T)
5. Coming home Ann thought that Richard thought she had got his message. (T)

6. Ann thinks that if Richard needs to find the sample wallpaper, he is going to look for it in the garage. (F)
7. While talking on the phone with Richard, the colleague thought that Richard thought that Ann thought that he was going to be at work on Friday. (T)

Supplementary Table 11

Narrative Texts used in Social Information Processing Manipulation for Art and Non-art Images in Study 2

Painting	Narrative text
Luncheon of the Boating Party by Pierre-Auguste Renoir	Back in Sussex, William couldn't wait to see friends he hadn't seen in years. He had days-worth of stories collected from his travels. The traditional luncheon was the perfect opportunity to reunite, he thought. But the moment he set down at the table, he felt great alienation. A visceral response, his body backed off the table, leaning against the railing. Voices were coming from a far. How proud, how disagreeable his friends were. As William listened to their stories, he was certain that his friends had never left this town. He answered with a smile, when he heard them calling his name, secretly knowing he will never see these people, who he knew once, ever again.
At Eternity's Gate by Vincent Van Gogh	The notice from the bank about his debt was the final straw. Daniel was weary of financial troubles, and now he was losing his house. He thought of gambling again. First a thrill, and then shame, sharply filled his body. He slammed his hands on his face, to hide the shame and punish the thrill. Who was to see him in the empty room? He was all alone. He yearned for someone to share the trouble with. He yearned for someone to have been there to keep him out of the trouble. He just didn’t see any way moving forward.
Flaming June by Frederic Leighton	Elena lived a life of imprisonment in a splendid house. When she stood on the balcony, those who looked up from the street envied her. Her life seemed perfect from a far, everyone thought it must be filled with unimaginable pleasures. Elena, on the other hand, would consider jumping off as she looked down from the balcony, to end the golden cage confinement. She was not allowed to leave the house, and would grow soulless as she waited for her captor. Falling asleep, Elena felt despair and anguish, yet she looked light and beautiful.
Woman Ironing by Pablo Picasso	Maria was so exhausted; she couldn’t bend her elbows. She couldn’t raise her neck, or move her arms. If she was to close her eyes for a moment, she would fall asleep right there. What if she sets the house on fire! Worse still, what if she burns her husband's shirt! These thoughts were startling, but her body stood still. No strength, yet racing thoughts, how heavy the chores weighed on her shoulders. She was going to wash the dishes after she finished ironing.
The Kiss by Gustav Klimt	Enveloped by his embrace, Grace melted away in her lover’s arms. She gently held his hand caressing her cheek, inciting him to lean in for the kiss. Leaving herself to his blissful embrace, she closed her eyes. She felt so safe, she could have fallen asleep right away. As his

	lips touched her cheek, Grace thought to herself, "Whatever our souls are made of, his and mine are the same."
The Absinthe Drinker by Pablo Picasso	“This is not a relapse”, Camila thought to herself. She wasn’t back on drinking. Why would her quitting prevent her from sitting down at this bar, the place she had her best memories? The place she had no memories of… “No! This is not a relapse!” she said to herself. All her friends were here. So what, if she didn’t know their names? Surely, at one drunken night or the other, they exchanged names. She just didn’t remember at this moment. This was not a relapse, no. Camilla would just take her usual order at her usual table in her usual bar, and think for a while.
The Man with the Hoe by Jean-François Millet	Mateo ran out of breath, so he rested on his hoe like a cane. He has been pushing himself hard these last few weeks, trying to plough the dry ground to sow seeds. Every day he has worked the field. The man was made weak by time and fate, but he was strong in will. He looked away, thinking back to his youth when this farm was fertile and lush with greenery. How he used to run through the tall crops… He looked backed down, it is all barren now. After catching his breath, he resumed ploughing again.
